# Supplementary material for: Reduced Expression of a Novel Midgut Trypsin Gene Involved in Protoxin Activation Correlates with Cry1Ac Resistance in a Laboratory-Selected Strain of Plutella xylostella (L.)
Source: Toxins (Basel). 2020 Jan 23;12(2):76. doi: 10.3390/toxins12020076 (PMC7076802; doi:10.3390/toxins12020076)
Supplement: Supplementary file 1 [file toxins-12-00076-s001.pdf]

Supplementary Materials: Reduced Expression of a Novel Midgut Trypsin Gene Involved in Protoxin Activation Correlates with Cry1Ac Resistance in a Laboratory-Selected Strain of *Plutella xylostella* (L.)

Lijun Gong, Shi Kang, Junlei Zhou, Dan Sun, Le Guo, Jianying Qin, Liuhong Zhu, Yang Bai, Fan Ye, Mazarin Akami, Qingjun Wu, Shaoli Wang, Baoyun Xu, Zhongxia Yang, Alejandra Bravo, Mario Soberón, Zhaojiang Guo *, Lizhang Wen * and Youjun Zhang *

Genes	PxTryp_SPc1	HaTryp_SPc	OtTryp_SPc3	DmTryp_SPc	AmTryp_SPc7	BtTryp_SPc1	ZnTryp_SPc	SgTryp_SPc	PhcTryp_SPc2
PxTryp_SPc1	100%								
HaTryp_SPc	52%	100%							
OtTryp_SPc3	35%	28%	100%						
DmTryp_SPc	26%	26%	41%	100%					
AmTryp_SPc7	29%	24%	30%	28%	100%				
BtTryp_SPc1	26%	23%	27%	27%	29%	100%			
ZnTryp_SPc	32%	26%	47%	39%	33%	31%	100%		
SgTryp_SPc	20%	25%	32%	25%	25%	18%	29%	100%	
PhcTryp_SPc2	24%	20%	26%	25%	29%	21%	23%	17%	100%

Sequence similarity

			<10%	>10%	>20%	>30%	>40%	>50%	100%

Figure S1. Pairwise comparisons of protein sequence identities among nine trypsin orthologs of PxTryp_SPc1 from different insect species. Abbreviations: PxTryp_SPc1 (*Plutella xylostella*, MN422356), HaTryp_SPc1 (*Helicoverpa armigera*, ABU98624), OtTryp_SPc3 (*Onthophagus taurus*, XP_022900611), DmTryp_SPc (*Drosophila melanogaster*, NP_001285772), AmTryp_SPc7 (*Apis mellifera*, XP_623564), BtTryp_SPc1 (*Bemisia tabaci*, XP_018896298), ZnTryp_SPc (*Zootermopsis nevadensis*, XP_021914447), SgTryp_SPc (*Schistocerca gregaria*, CAA70820) and PhcTryp_SPc2 (*Pediculus humanus corporis*, AAV48634).

Table 1. List of primers used in this study.

Purpose	Primer Name	Primer Sequence (5′–3′)	Tm (°C)	PCR Product Size (bp)	Positions (bp)[†]
Gene cloning	Try1-F	GGTTACCTGGGCTGTGCTTC	65	799	-2–766
	Try1-R	CTCATTCGTCGGTTTGGCTT			
qPCR analysis	qTry1-F	GAATGTCAGCACCAGCAGC	57	135	217–351

	qTry1-R	CAGATGTCGGCGTCGTAG			
	qL32-F	CCAATTTACCGCCCTACC	57	120	-
	qL32-R	TACCCTGTTGTCAATACCTCT			
dsRNA synthesis‡	dsTry1-F	GGCTGTGCTTCTTCTTACCG	58	389	10–399
	dsTry1-R	GCTGGATGGCAGGAGTGA			
	dsEGFP-F	T7-CCACAAGTTCAGCGTGTCCG	58	469	-
	dsEGFP-R	T7-AAGTTCACCTTGATGCCGTTC			

†Positions corresponding to the full-length cDNA sequence (GenBank accession no. MN422356) of the *PxTryp_SPc1* gene. ‡T7 RNA polymerase promoter sequence (TAATACGACTCACTATAGGGAGA) was appended to the forward and reverse primers 5′ ends to synthesize dsRNA template. The short line “-” denotes Not Available (N/A).
